# Aged Residential Care Health Utilisation Study (ARCHUS): a randomised controlled trial to reduce acute hospitalisations from residential aged care

**DOI:** 10.1186/1471-2318-12-54

**Published:** 2012-09-13

**Authors:** Susan J Foster, Michal Boyd, Joanna B Broad, Noeline Whitehead, Ngaire Kerse, Thomas Lumley, Martin J Connolly

**Affiliations:** 1 Freemasons’ Department of Geriatric Medicine, University of Auckland, C/- WDHB, Private Bag 93503, Takapuna, Auckland, 0740, New Zealand; 2 Counties Manukau District Health Board, Auckland, New Zealand; 3 General Practice and Primary Health Care, School of Population Health, University of Auckland, Auckland, New Zealand; 4 Department of Statistics, University of Auckland, Auckland, New Zealand

## Abstract

**Background:**

For residents of long term care, hospitalisations can cause distress and disruption, and often result in further medical complications. Multi-disciplinary team interventions have been shown to improve the health of Residential Aged Care (RAC) residents, decreasing the need for acute hospitalisation, yet there are few randomised controlled trials of these complex interventions. This paper describes a randomised controlled trial of a structured multi-disciplinary team and gerontology nurse specialist (GNS) intervention aiming to reduce residents’ avoidable hospitalisations.

**Methods/Design:**

This Aged Residential Care Healthcare Utilisation Study (ARCHUS) is a cluster- randomised controlled trial (n = 1700 residents) of a complex multi-disciplinary team intervention in long-term care facilities. Eligible facilities certified for residential care were selected from those identified as at moderate or higher risk of resident potentially avoidable hospitalisations by statistical modelling. The facilities were all located in the Auckland region, New Zealand and were stratified by District Health Board (DHB).

**Intervention:**

The intervention provided a structured GNS intervention including a baseline facility needs assessment, quality indicator benchmarking, a staff education programme and care coordination. Alongside this, three multi-disciplinary team (MDT) meetings were held involving a geriatrician, facility GP, pharmacist, GNS and senior nursing staff.

**Outcomes:**

Hospitalisations are recorded from routinely-collected acute admissions during the 9-month intervention period followed by a 5-month follow-up period. ICD diagnosis codes are used in a pre-specified definition of potentially reducible admissions.

**Discussion:**

This randomised-controlled trial will evaluate a complex intervention to increase early identification and intervention to improve the health of residents of long term care. The results of this trial are expected in early 2013.

**Trial registration:**

Australian New Zealand Clinical Trials Registry: ACTRN 12611000187943

## Background

Older people’s health is increasingly important for New Zealand. Mid-range projections from Statistics New Zealand show the proportion of the population aged 75+ will increase from 5.6% in 2006, to 10.4% by 2031 and 14.7% by 2051. Similar ageing demographics exist throughout the OECD, though New Zealand is still ‘young’ in comparison to most OECD nations. Those over 85 years of age comprised 1.3% of the population in 2001, but used half of residential aged care (RAC) costs [1]. New Zealand has high rates of use of residential aged care ( [2], in press) with about 70% of residents receiving public funds for their care (Grant Thornton 2010). Healthcare spending will increase markedly with the rise of these ‘oldest old’.

In 2008 a census of older people in residential care was completed in the Auckland region in New Zealand. The findings revealed that 28% of those aged 85+ reside in RAC facilities, and residents were increasingly older and more dependent than previous surveys (2003, 1995, 1989) [3,4]. The need for residential care does not appear to have diminished even though a number of initiatives including Ageing-in-Place have been implemented [5]. Those living in residential care facilities are at increased risk of hospitalisation because their physical and mental frailty increases their susceptibility to acute illness and injuries [6]. Improvements in quality indicators such as urinary tract infections, wounds, and falls are associated with better resident health and decreased hospitalisations [7,8]. Quality of care depends on the staff, the facility and services available (Schnelle 2004). Residential aged care needs to be ‘better managed’ to improve quality [9] and reduce costs. ARC staff turnover is high and increasing, with high vacancy rates, particularly for registered nurses and caregivers [10]. There is a need to develop new ways to support the RAC industry that improve resident outcomes, which in turn possibly reduce avoidable hospitalisations.

Flicker [9] made recommendations for improving RAC care: an interdisciplinary team approach, and partnering with tertiary institutions and expert groups to develop clinical guidelines to promote best practice. Several resident and facility interventions have been proven to improve health care outcomes [11]. These include interdisciplinary team medication reviews [12], nutritional screening and intervention [13], facilitated end of life advanced care planning [14] and outreach education and clinical coaching [15].

Ambulatory sensitive hospitalisations (ASH) are those that are potentially avoidable through early primary care intervention, and include diagnoses such as syncope, congestive heart failure (CHF), constipation, dehydration and volume depletion, fall and hip fracture, influenza, pneumonia and urinary tract infection (UTI) [16]. In a New Zealand review of interventions specifically to reduce Ambulatory Sensitive Hospitalisations in younger people [16] reported evidence for successful strategies including: inter-disciplinary, collaborative and patient-centred team approaches, education-based comprehensive care programmes, outreach collaborations by both specialists and generalists for disease-specific interventions and increased access to services for all including the under-served. However residential aged care was not the focus for this review and most reported trials were single-disease based. There is a large gap in the knowledge related to residential aged care transfers to acute hospitals and how best to reduce them. There is a need for detailed examination of available data to better understand why hospitalisations occur in New Zealand settings, in order to intervene rationally.

Evidence supports new models of care in RAC result in reduced hospitalisations. Large scale organisations such as Evercare, a large RCT of Nurse Practitioner input into RAC in the United States, reduced acute hospitalisations [17] and similar results were reported from Canada [18]. However the scope of these large scale model changes is often beyond local funding capacity and advanced nursing capability. In addition these studies and their subsequent clinical applications have provided only limited understanding of the types of admissions impacted. It is clear that some admissions are needed for acute and specialist care; however evidence suggests that better preventive care and emphasis on managing acute illness in situ improves resident health outcomes. [19,20]. Structured RAC interventions have demonstrated success in functional outcomes [21] and falls [22], but apart from large scale model change, actual reductions in hospitalisations are not yet proven.

## Methods/design

This is a randomised controlled trial of a complex intervention of a package of supports and services provided by District Health Boards (DHBs) interdisciplinary team outreach to RAC facilities.

The study is taking place in the greater Auckland area of New Zealand. Auckland is the largest metropolitan area of New Zealand with a population of 1.4 million people. Within this region there are 175 aged care facilities, from which the participating facilities were selected on the basis of higher than ‘expected’ hospital admission rates. A total sample size of 1400 resident-years was originally anticipated to provide 80% power (5% significance) to detect a 25% reduction in rate of ASH hospitalisations in the intervention group compared to the control group when an event rate of 60 events per 100 years was expected. However, observed event rate in another cohort after commencement of the trial showed a lower event rate, so power estimates were recalculated. With 18 facilities for each group each with 14 months of follow-up, an average of 38 beds per facility and bed occupancy of 90%, we expect a total of 1500 resident years of follow-up. Revised power is estimated at 53%, considering:

• Inflated sample size as the design effect of 2.0 will allow for moderate intracluster correlation for hospitalisation rates of 0.025 [23,24]

• Rate of 35 ASH admissions per 100 resident years in control facilities vs. 26 ASH admissions in intervention facilities, assuming a Poisson distribution where the mean equals the variance. The control event rate was estimated from re-analysis of the RACIP study (Boyd et al., 2008) and the OPAL study cohort (results not yet published).

However, we anticipate an improvement in power because;

• the facilities were chosen (from statistical modelling) for their higher event rates (e.g. an event rate of 0.40 would provide power of 0.67)

• short-stay residents (under-represented in OPAL-based rates) have a higher event rate

• adjustment for covariates in our analysis will reduce confidence intervals around effect size

### Facility selection

Eligible facilities were all those certified by the DHBs as providing long-term care in the region. Details were obtained of bed numbers and care level provided. Prior to facility recruitment, we examined structural aspects of RAC and evaluated potential associations with avoidable hospitalisations, known as ambulatory sensitive hospitalisations (ASH), taking into account individuals’ demographics and health status. Routine reporting of ASH events in New Zealand ceases at age 75, partly because established classifications of ASH diagnoses are perceived to be ‘less relevant’ for older people, particularly those in RAC. We thus developed an ASH classification based on diagnoses that were relevant for the RAC population. This classification method was used to identify facilities with high hospitalisation rates using multivariate modelling techniques that will be reported separately. Facilities were selected from those identified as at moderate or greater risk of resident hospitalisation based on factors identified during modelling.

### Facility recruitment

**Figure 1:**
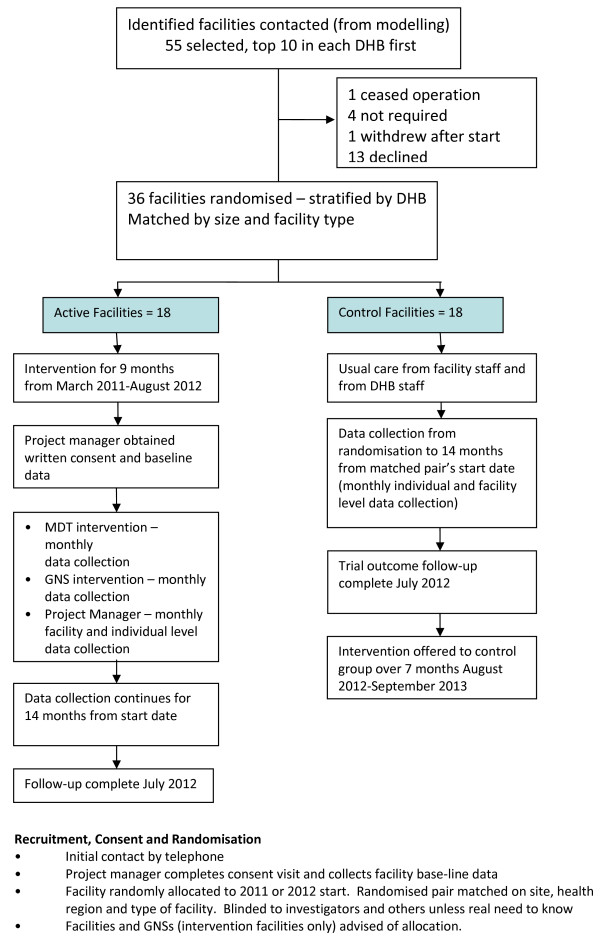
**Process for recruitment and randomisation of facilities**.

Initially facilities were contacted by phone by the project manager and invited to participate in the study. This was followed by a visit to confirm written informed consent and to obtain base-line facility data after which the facility was advised whether allocated to intervention or control. In all, a total of 50 facilities were contacted and invited to participate. Twelve facilities declined to take part, citing work load and the similarity to another study recently undertaken as the main reasons. Two facilities withdrew shortly after randomisation because they did not desire to continue with the research project. These facilities were replaced with two other facilities as the intervention had not begun. One control facility has withdrawn half way through the study as it had changed ownership and the new owners did not want to continue participation in the research. Overall there has been an very positive response to the study as most of the facilities are keen to receive the perceived benefits of the intervention (Figure 1).

### Randomisation

Thirty-six facilities from three Auckland DHBs were randomised following consent. Facility randomisation was conducted by random number allocation, stratified by DHB and mix of care types (rest home only, or a mix of rest home, dementia and hospital beds). Facility identification was blinded to the main investigators wherever possible (some of the researchers were involved in clinical aspects of the intervention so were required to know only those facilities to which they provided clinical input). The control group were offered the same interventions following the completion of the intervention stage of the study. Control facilities were all blinded to all investigators.

### Ethical considerations

Full ethics approval was given by the Northern Y Ethics Committee in January 2011 (NTY 10/11/090). Consent was obtained for facility participation rather than individual resident participation, as no identifying data is being collected and none of the interventions will be outside the usual clinical practice provided by facility staff. Only National Health Index (NHI) numbers (a national healthcare identification number for each person in New Zealand) were collected by the researchers. All individual residents’ information was kept anonymous to the researchers.

### Intervention

The intervention builds on aged care programmes already in place in the three Auckland region DHBs. Each DHB has gerontology clinical nurse specialists (GNSs) providing outreach to facilities, but the models of care vary between the DHBs and there is no consistent method to Identify high-risk facilities. The trial intervention delivers outreach tailored to facility needs. The multi-disciplinary team is considered a critical element [25]. These teams were established at each of the three Auckland DHBs, building on existing RAC outreach services. The team comprises the facility General Medical Practitioner and Nurse Manager, a DHB geriatrician, a DHB Gerontology Nurse Specialist and a community pharmacist that services the facility or DHB clinical pharmacist.

The interventions in this study include:

• Initial baseline facility assessment to identify areas of need and facility care plan

• Benchmarking monitoring resident quality indicators that are linked to the quality of care provided (falls, nutrition, use of restraints, weight loss, UTIs, residents on nine or more medications).

• Three one-hour multi-disciplinary team (MDT) meetings to be held monthly for the first three months at each intervention facility, including medication reviews by the geriatrician in conjunction with the GP, pharmacist and nurse manager. At most, six residents were considered at each meeting with new admissions, those recently hospitalised, and those residents on nine or more medications given priority.

• Gerontology education and clinical coaching for RAC nurses and caregivers including advanced care planning for end-of-life care, nutrition and hydration, early identification of illness, falls prevention, end-stage dementia care, communication with families and practical aspects concerned with resident care.

The intervention continued for nine months with the intensity of the intervention decreasing over time to foster facility independence prior to the conclusion of active involvement, including months 6 and 8 where facilities did not receive any input by the GNSs. The GNSs began the intervention with one new facility per month in order to allow sufficient time for the organisation and delivery of the intervention.

Facilities provided monthly lists of all residents showing only residents’ unique identifiers, care type, and admission and discharge dates, to facilitate tracking residents and for sub-group analyses. The data for all acute hospitalisations and deaths will be retrieved from the Ministry of Health’s routinely collected public hospital admissions data on presentation of the unique identifier.

### Outcome measures

The primary outcome measurements comprise:

 1. Rate of ASH admissions classified using a pre-determined set of ICD codes.

 2. Number of acute admission hospital days.

 3. Number of deaths from any cause, including death in acute hospital or elsewhere obtained from a national mortality database.

Numbers and rates for these three outcomes will be calculated as the proportion of residents hospitalised (1), number of hospital days per occupied bed per year (2) and deaths per person-year (3). Sub-group analyses will be conducted, by age group and gender, and by facility type.

This data will be collected from the date of randomisation to 14 months after the first visit by the GNS in the intervention arm, and for the paired facility in the control arm. Hospitalisation records will be sought five months after the final facility completes its intervention, in order to allow for any delays in registering admissions. Paper reports from each participating facility will enable checking against national records.

### Analyses

Analysis of primary endpoints will compare rate of ASH admissions between treatment groups followed by other main endpoints. Simple unadjusted rates, relative risks and 95% confidence intervals will be obtained initially, with subsequent multiple regression analysis adjusting for paired randomisation and other variables. Negative binomial regression will be used. Baseline rates will likely be included as a covariate in regression models as pre-intervention hospitalisation rates will be highly predictive of post-intervention rates.

Sub-group analyses to check for effects that differ from the overall treatment effect will be performed for the following:

• facilities classed as charitable, religious or welfare facilities vs. for “for profit” facilities

• for facilities that have only rest-home beds vs. those with both rest-home and hospital beds

• for long-stay vs. short-term (e.g. respite or palliative care) residents

• for facilities providing after-hours primary care cover through either their usual GP or a contracted after-hours primary care provider, vs. those without such cover (effectively using ambulance and emergency department for after-hours primary care)

All main analyses will be formally analysed on an "intention to treat" basis. Tests of significance will be two-tailed. Analysis of secondary outcomes will use standard statistical procedures applicable to categorical, continuous, or failure-time data as appropriate.

## Discussion

This study is designed to provide an intervention to support clinical decisions about at-risk RAC residents and to improve quality of care. The aim is to increase early identification of potential health issues thus improving resident health and reducing avoidable emergency department presentations and hospital admissions. It will inform appropriate, cost-effective use of acute services and will promote access to proven interventions and practice-orientated decision making. It is intended that this study will impact on policy and on planning and financing models of residential aged care, and will increase continuity of care between residential care, primary and secondary care.

This project has potential to lead to the redesign of systems and also to refine policies such as care practices, staffing levels and patterns (night-staffing, after-hours medical care and advanced nursing interventions). It is also hoped that the study will facilitate:

• improved integration of RAC with geriatricians and with emergency/acute services;

• clinical education for RAC staff in facilities to increase their use of research and current guidelines;

• improved RAC palliative care practices;

• alternative residential aged care models to provide targeted care for high risk groups, e.g.: those with end-stage dementia

Other new models may include short RAC stays for rehabilitation and aged care community integration. Reducing hospitalisations will benefit older people residing in RAC because acute hospitalisation may increase the risk of disability and death [26]. There is evidence that where treatment is offered in situ, residents recover more quickly, reducing confusion and deterioration [14,27].

The issues that this trial addresses are not unique to Auckland or indeed to New Zealand. Identification and targeting of high admission facilities and refinement of proven interventions directed toward those facilities and modified individually in situ, means that the results of this RCT are likely to be widely applicable internationally.

## Competing interests

The authors declare that they have no competing interests.

## Authors’ contributions

**SF** is the main author of the paper and is the project manager of the study. **MB** is a co-investigator on the ARCHUS study, conceived the study and developed the interventions, and provided critical comment to the manuscript. **JBB** is an epidemiologist, participated in the design of the study, is responsible for the data analyses, and contributed to the draft of the paper. **NW** advised on the nursing interventions and provided critical comment to the manuscript. **NK** is a co-investigator, an academic GP who participated in the design of the study. **TL** is a statistician and co-investigator and provided statistical advice. **MC** is a geriatrician and the principal investigator of the study and was responsible for its design and general oversight. All authors have read and approved the final manuscript.

## Pre-publication history

The pre-publication history for this paper can be accessed here:

http://www.biomedcentral.com/1471-2318/12/54/prepub
